# Coronavirus Disease Stress Among Italian Healthcare Workers: The Role of Coping Humor

**DOI:** 10.3389/fpsyg.2020.601574

**Published:** 2021-01-25

**Authors:** Carla Canestrari, Ramona Bongelli, Alessandra Fermani, Ilaria Riccioni, Alessia Bertolazzi, Morena Muzi, Roberto Burro

**Affiliations:** ^1^Department of Education, Cultural Heritage and Tourism, University of Macerata, Macerata, Italy; ^2^Department of Political Science, Communication, International Relations, University of Macerata, Macerata, Italy; ^3^Department of Human Sciences, University of Verona, Verona, Italy

**Keywords:** coping, humor, perceived stress, COVID-19, healthcare workers, psychological distance

## Abstract

The study aimed to understand how coping strategies in general and humor-based coping strategies in particular modulate the perception of pandemic-related stress in a sample of Italian healthcare workers during the coronavirus disease (COVID-19) outbreak in Italy. A total of 625 healthcare workers anonymously and voluntarily completed a 10-min questionnaire, which included psychometrically valid measurements preceded by a set of questions aimed at determining workers’ exposure to COVID-19. The Perceived Stress Scale was used to measure healthcare workers’ stress levels, and the Brief COPE Scale and Coping Humor Scale were used to assess participants’ avoidant or approach coping style and verify the degree to which they relied on humor to cope with stress. The results show that (1) levels of perceived stress were higher in healthcare workers who were more exposed to COVID-19 (i.e., who came into contact with COVID-19 patients or worked in wards dedicated to COVID-19) in comparison to less-exposed workers; (2) participants who reported a higher use of avoidant coping strategies perceived the situation as more stressful than those who used them less; and (3) healthcare workers who reported higher use of humor-based coping strategies perceived the situation as less stressful in comparison with those who reported less use of coping humor. Such findings expanded other research studies by including coping humor as a potential factor to mitigate the perceived stress related to COVID-19. The paper concludes with a discussion of implications for future research and limitations of the study.

## Introduction

The coronavirus disease (COVID-19), a highly contagious respiratory disease caused by a newly discovered virus (SARS-CoV-2), was first reported in December 2019 in Wuhan, China (Chan et al., 2020); it rapidly spread across the globe and was declared a pandemic by the World Health Organization (WHO) in March 2020. So far, the confirmed number of persons infected and deceased due to COVID-19 has exceeded 57 million and 1.3 million, respectively, worldwide^1^. The outbreak of the COVID-19 pandemic negatively impacted clinical and risk management in healthcare systems and posed great challenges to healthcare workers (HCWs) worldwide, particularly in countries such as Italy, where COVID-19 greatly impacted public health. In Italy, the first patients were diagnosed on January 30, 2020.^2^ The Italian government declared a state of emergency on January 31, and imposed a lockdown on March 4.^3^ How stressful Italian HCWs perceived the situation at work and what psychological strategies they used to cope against the perceived stress during the first wave of the COVID-19 pandemic in Italy are at issue in the present study.

### COVID-19, Stress of HCWs, and Coping Strategies

There is evidence that this outbreak has impacted the psychological wellbeing of HCWs in several ways, causing anxiety (Ahn et al., 2020; Magnavita et al., 2020; Preti et al., 2020; Raccanello et al., 2020; Xiao et al., 2020), burnout (Denning et al., 2020), depression (Lam et al., 2020; Preti et al., 2020), insomnia (Magnavita et al., 2020; Preti et al., 2020; Stojanov et al., 2020; Xiao et al., 2020), and emotional dysregulation (Ornell et al., 2020). HCWs have been forced to cope with extraordinary challenges and perceived stress by relying on coping strategies and positive psychological resources, as they did during previous epidemics (e.g., Wu et al., 2009; Park et al., 2018; Preti et al., 2020). For example, hardiness was a protective factor for nurses’ mental health during the MERS-CoV epidemic in 2015 (Park et al., 2018), and vigor and perceived organizational support protected nurses from burnout during the SARS epidemic in 2004 (Marjanović et al., 2007). Regarding COVID-19, resilience, adaptive coping strategies, and social support helped alleviate disease-related stressful experiences and acute stress disorders in Chinese students (Ye et al., 2020). Social support reduced Chinese medical staff’s anxiety and stress and positively affected their self-efficacy (Xiao et al., 2020). In addition, psychological distancing (in terms of fear of being infected, infecting relatives and friends, working into contact with COVID-19 patients, and so on) plays a significant role in protecting HCWs mental health and wellbeing (Lam et al., 2020). However, only a few studies have investigated HCWs’ positive psychological resources in times of COVID-19, including coping and adaptive strategies (Preti et al., 2020).

### Stress and Coping Humor

According to Vaillant (2002, p. 63), humor, early defined as a defense mechanism by Freud (1928), is a mature defense namely an adaptive coping strategy that “permits the expression of emotion without individual discomfort and without unpleasant effects upon others.” Among the character of strengths pointed out in the frame of positive psychology, humor (in terms of liking to laugh and joke and bringing smiles to other people) plays a significant role in coping with stressful events (e.g., Vaillant, 2000; Peterson and Seligman, 2004; Müller and Ruch, 2011), contributes to resiliency, is a significant predictor of wellbeing and satisfaction (Geisler and Weber, 2010; Kuiper, 2012), and is negatively related to depressive symptoms in response to stress (Martin and Lefcourt, 1983).

Given that the cognitive process activated for understanding humor elicits frame shifting (Koestler, 1964; Wyer and Collins, 1992), humorously depicting a problematic situation may result in its reappraisal, thereby positively changing the perspective. In other words, a representational change is needed to resolve the incongruity in understanding humor (Forabosco, 1992). A cognitive reorganization of the initial representation is pivotal both in understanding humor and solving problems (e.g., Gick and Lockhart, 1995; Kozbelt and Nishioka, 2010; Canestrari et al., 2018), since it involves overcoming fixities that may arise from considering the situation only from the salient and early perspective. The same cognitive process of shifting away from the initial representation of a problematic situation and overcoming its fixed representation applies to coping with it humorously (Martin, 1989). Approaching a perceived stressful situation with humor positively impacts emotion regulation, cognitive appraisal, and reappraisal of the problematic situation (e.g., Martin and Lefcourt, 1983; Martin et al., 1993; Abel, 2002; Geisler and Weber, 2010; Doosje et al., 2012; Kuiper, 2012). Humor as a protective factor for wellbeing has been verified in several contexts, ranging from clinical settings (e.g., Ventis et al., 2001; Svebak et al., 2006; Dionigi and Canestrari, 2016, 2018a,b) to daily hassles (e.g., Martin and Lefcourt, 1983; Martin et al., 1993; Abel, 2002; Kuiper, 2012 for an overview).

Despite the evidence, HCWs’ use of humor as a coping strategy against stressful situations, including pandemics, is still a poorly investigated topic. To the best of our knowledge, till date, only one study, conducted on a sample of Israeli nursing students during the COVID-19 pandemic, showed that the usage of humor as a coping strategy was associated (together with resilience and high level of self-esteem) with significantly lower anxiety levels (Savitsky et al., 2020). This result agrees with the above-mentioned literature on the protective and coping functions of humor and paves the way for investigation of a significant, albeit neglected, issue.

### Aim of the Present Study

To contribute to a better understanding of the coping strategies, including humor, used by HCWs to face COVID-19-related stress, we surveyed a sample of Italian HCWs during the outbreak of COVID-19 in Italy. We considered their exposure to COVID-19-related risks (i.e., working in COVID wards or having come into contact with COVID-19 patients), levels of perceived stress, adaptive and maladaptive coping strategies, and usage of humor as a coping strategy. Moreover, we verified whether their levels of perceived stress were associated with adaptive or maladaptive coping strategies and to what extent they have been relying on humor to face COVID-19-related stress. We hypothesized that exposure to infection risk is associated with an increasing level of perceived stress and that the use of humor as a coping strategy is negatively associated against perceived stress.

## Materials and Methods

### Study Design and Participants

A web-based survey was carried out among Italian HCWs from May 15 to July 30, 2020.

Participants who met the following criteria were included: (1) Italian HCWs (physicians and nurses) working in hospitals or primary care services, (2) physicians and nurses enrolled in professional orders and/or associations, (3) HCWs who volunteered for the survey, and (4) those who completed the questionnaire only once, on the basis of IP-based duplicate protection, which allows one response per IP address.

Exclusion criteria included: (1) the person being unable to understand the questionnaire and (2) the person not being a physician or nurse.

To carry out the survey, first, the request for survey participation was sent by email to all medical and nursing professional orders in Italy and to the main professional associations of both physicians and nurses. The request, in addition to submitting the research protocol, asked organizations to send the link to the self-administered questionnaire to their members or, alternatively, publish the link on their website. After 3 weeks, the professional organizations that did not respond to the first request were sent the participation request again.

The survey was conducted through LimeSurvey software on a LAMP (Linux, Apache, MySQL, PHP) server, a common example of a web service stack. All the communication was encrypted, using Hyper Text Transfer Protocol Secure (HTTPS) and Secure Sockets Layer (SSL). The questionnaire opened with an informed consent form, which explained the aim of the study and assured participants’ anonymity. It comprised 10 questions about respondents’ personal and professional characteristics and three psychometric validated scales described below (see section “Measures”). All questions were mandatory, and the average time allotted for filling the questionnaire was 10 min.

We obtained a sample size of 625 participants (142 males and 483 females). The age ranged from 21 to 81 years (*M* = 44.39; SD = 11.91). To describe the sample, we measured some socio-demographic characteristics concerning private and professional life: marital status; relationship with religion; familial status; profession (physician or nurse); area of profession (recoded as: medical specializations, surgical specializations, clinical services area, and primary care nursing services); job location in Italy (recoded as: north, center, and south); length of service; exposure to COVID-19, in terms of having worked in COVID wards, having come into contact with COVID-19 patients, and neither having come into contact with COVID-19 patients nor having worked in wards dedicated to them (recoded respectively as follows: yes/no, contact/no contact, yes/no); have been swabbed during COVID-19 or not; and contracted the virus or not.

Descriptive statistics are displayed in Table 1.

**TABLE 1:** Descriptive statistics: frequencies (%).

**Variables**	**Males (%)**	**Females (%)**	**Total (%)**
***Marital status***			
Never married	35 (5.6)	136 (21.8)	171 (27.4)
Domestic partner	23 (3.7)	81 (13.0)	104 (16.6)
Divorced/separated	11 (1.8)	49 (7.8)	60 (9.6)
Married	72 (11.5)	207 (33.1)	279 (44.6)
Widower/widow	1 (0.2)	10 (1.6)	11 (1.8)
***Children***			
Yes	87 (13.9)	278 (44.5)	365 (58.4)
No	55 (8.8)	205 (32.8)	260 (41.6)
***Religion***			
Non-believer	32 (5.2)	69 (11)	101 (16.2)
Prefer not to answer	7 (1.1)	27 (4.3)	34 (5.4)
Occasional practitioner	45 (7.2)	195 (31.2)	240 (38.4)
Regular practitioner	18 (2.9)	82 (13.1)	100 (16)
Non-practicing believer	40 (6.4)	110 (17.6)	150 (24)
***Job position***			
Physician	54 (8.6)	101 (16.2)	155 (24.8)
Nurse	88 (14.1)	382 (61.1)	470 (75.2)
***Geographical area***			
North	95 (15.2)	343 (54.9)	438 (70.1)
Center	23 (3.7)	97 (15.5)	120 (19.2)
South	24 (3.8)	43 (6.9)	67 (10.7)
***Job professional area***			
Medical specializations	77 (12.3)	249 (39.8)	326 (52.2)
Surgical specializations	22 (3.5)	75 (12.0)	97 (15.5)
Clinical services	25 (4.0)	107 (17.1)	132 (21.1)
Primary care nursing services	18 (2.9)	52 (8.3)	70 (11.2)
***Seniority***			
More than 20 years	74 (11.8)	222 (35.5)	296 (47.4)
10–20 years	26 (4.2)	88 (14.1)	114 (18.2)
5–10 years	16 (2.6)	64 (10.2)	80 (12.8)
Less than 5 years	26 (4.2)	109 (17.4)	135 (21.6)
***Worked with COVID-19 patients***			
Yes	59 (9.4)	194 (31.0)	253 (40.5)
No	35 (5.6)	134 (21.4)	169 (27.0)
Contact	48 (7.7)	155 (24.8)	203 (32.5)
***Test***			
No	63 (10.1)	204 (32.6)	267 (42.7)
Yes	79 (12.6)	279 (44.6)	358 (57.3)
***COVID-19***			
Yes	9 (1.4)	45 (7.2)	54 (8.6)
No	113 (18.1)	375 (60.0)	488 (78.1)
Perhaps	20 (3.2)	63 (10.1)	83 (13.3)

### Measures

The Italian health professionals’ perceived stress was measured using the Italian Perceived Stress Scale-10 (IPSS-10), the Italian version (Fossati, 2010) of the Perceived Stress Scale (PSS). PSS is the most widely used instrument for measuring the perception of stress. Its original version consisted of 14 items (Cohen et al., 1983), but the version used the most comprises10 items; the 10-item version is preferred since it presents a slightly better internal consistency (Cohen and Williamson, 1988; Mondo et al., 2019). PSS measures “the degree to which situations in one’s life are appraised as stressful. Items were designed to tap how unpredictable, uncontrollable, and overloaded respondents find their lives” (Cohen et al., 1994, p. 1). The items are easy to understand, of a general nature, relatively free of content specificity, and ask about feelings and thoughts during the last month. The response alternatives are also simple to understand. Respondents are asked how often they felt a certain way. Sample items are (3 and 8, respectively) “In the last month, how often have you felt nervous and stressed?” (*Nell’ultimo mese, con che frequenza si eÌ sentito nervoso o “stressato”?*); “In the last month, how often have you felt that you were on top of things?” (*Nell’ultimo mese, con che frequenza ha sentito di padroneggiare la situazione?*). All the items can be answered on a five-point Likert scale: 0 = never, 1 = almost never, 2 = sometimes, 3 = fairly often, and 4 = very often. Cronbach’s alpha for the IPSS was 0.885, and McDonald’s omega was 0.888.

The Italian health professionals’ coping strategies were evaluated using the Brief*-*COPE (Carver, 1997), the short version of the original 60-item COPE (Coping Orientation to Problems Experienced) inventory (Carver et al., 1989). The Brief-COPE is one of the most widely used self-report questionnaires in health contexts, and although it has been prevalently applied to measure patients’ coping strategies, it has been also applied—with good results—for measuring doctors’ and nurses’ coping strategies, as Mache (2012) claims. It consists of 14 facet scales (each comprising two items), which represent 14 different coping strategies, some adaptive, others problematic or ineffective (Carver, 1997; Carver and Scheier, 1998): active coping, planning, positive reframing, acceptance, seeking emotional support, seeking instrumental support, self-distraction, denial, venting, substance use, behavioral disengagement, self-blame, humor, and religion. The facet scales can be grouped into two overarching factors: approach (active coping, planning, positive reframing, acceptance, seeking emotional support, and seeking instrumental support) and avoidant coping styles (self-distraction, denial, venting, substance use, behavioral disengagement, and self-blame). Humor and religion facets belong neither to approach nor to avoidance coping, since they consist of both adaptive and problematic components (Eisenberg et al., 2012).

The 28 items are measured using scores ranging from 0 (I have not been doing this at all) to 3 (I have been doing this a lot). We adapted the original Brief-COPE scale into Italian using a forward and backward translation process to guarantee correspondence between the Italian and original English versions.^4^ Since we wanted to measure the Italian healthcare professionals’ situational and retrospective coping strategies in relation to a specific stressful circumstance (COVID-19 pandemic), items were expressed in the past tense. According to Carver (1997, pp. 95–98), items can indeed “be converted to a dispositional ‘coping style’ format […] or a situational concurrent format, by changing verb forms […]. They can assume a retrospective, situational format […], a concurrent situational format […] or even a dispositional format”.

Sample items are “I’ve been concentrating my efforts on doing something about the situation I’m in” (*Ho concentrato i miei sforzi per fare qualcosa per la situazione in cui mi trovavo*) (Approach-item 6); “I’ve been saying to myself ‘this isn’t real”’ (*Mi sono detto/a: “questo non è reale”*) (Avoidant-item 3); “I’ve been making jokes about it” (*Ho scherzato sulla situazione*) (Humor-item 18); and “I’ve been praying or meditating” (*Ho pregato o meditato*) (Religion-item 27).

We performed a confirmatory factor analysis (CFA) to test the goodness of fit of the factor structure of the Brief-COPE Italian version using the comparative fit index (CFI), the Tucker–Lewis index (TLI), the root mean square error of approximation (RMSEA), and the standardized root mean square residual (SRMR), with CFI and TLI ≥ 0.90, RMSEA ≤ 0.08, and SRMR ≤ 0.10 as threshold values (Kline, 2016). The outcomes of the CFA supported the hypothesized structure. All standardized factor loadings were statistically significant (with values between 0.430 and 0.989), and the goodness of fit indexes were acceptable (CFI = 0.927; TLI = 0.919; RMSEA = 0.078; SRMR = 0.089).

Cronbach’s alpha for the Brief-COPE was 0.811, and McDonald’s omega was 0.774. Specifically, for the avoiding strategies, the alpha was 0.771 and the omega was 0.769; for the approaching coping strategies, the alpha was 0.820 and the omega was 0.805; for the humor coping strategies, the alpha was 0.862; for the religion coping strategies, the alpha was 0.846 (in the last two cases, the omega was not reported because humor and religion coping strategies only had two items each).

The Italian health professionals’ coping humor was measured using an Italian version of the Coping Humor Scale (CHS; Martin and Lefcourt, 1983). The CHS is a seven-item inventory with a four-point Likert scale (ranging from one to four), translated in many languages, which measures “the degree to which respondents make use of humor in coping with stress in their lives” (Martin, 1996, p. 252). For its Italian translation, we applied the forward and backward translation process, as we did for the Brief-COPE.

Sample items are (1 and 6, respectively) “I often lose my sense of humor when I am having problems” (*Spesso perdo il senso dell’umorismo quando ho problemi*) and “I can usually find something to laugh or joke about even in trying situations” (*Di solito riesco a trovare qualcosa su cui ridere o scherzare persino in situazioni difficili*).

Moreover, in this case, a CFA was conducted to test the goodness of fit of the factor structure of the Italian CHS version. As shown in Martin (1996), item 4 had insufficient psychometric properties (the standardized factor loading was equal to −0.043 and *p*-value = 0.263); therefore, it was excluded. The performance of the CHS in Italian based on the remaining six-items was very good (CFI = 0.994; TLI = 0.990; RMSEA = 0.080; SRMR = 0.046). All standardized factor loadings were statistically significant (with values between 0.514 and 0.878).

Cronbach’s alpha for the CHS was 0.812, and McDonald’s omega was 0.821.

### Data Analysis

To verify whether stress perceived among HCWs due to COVID-19 was related to their exposure to COVID-19 patients, coping strategies, and coping humor, we studied the relationship between health professionals’ coping strategies (avoidant, approach, humor, and religion; based on Brief-COPE scores) and their coping humor (based on CHS score) on perceived stress (based on IPSS-10 score). We also considered whether the health professionals worked with COVID-19 patients in a dedicated ward (options: Yes/Not in a dedicated ward, but there were contacts with patients/No) and if they contracted the virus (options: Yes/Perhaps/No).

For the data analysis, we used the R software environment for statistical computing and graphics, version 4.0.0 (R Core Team, 2020). Additional packages are as follows: emmeans (Lenth, 2020), effects and car (Fox and Weisberg, 2019), reshape (Wickham, 2007), lavaan (Rosseel, 2012), semTools (Jorgensen et al., 2020), performance (Lüdecke et al., 2020), Rmisc (Hope, 2013). Descriptive statistical analyses (*n*, %) were performed using cross-tabulation. Descriptive statistics and correlations among all used scales were performed (see Table 2).

**TABLE 2:** Descriptive statistics and correlations among all used scales.

**Descriptive statistics**								

**Variable**	***M***	**SD**	**1**	**2**	**3**	**4**	**5**	**6**
1. Stress	21.002	8.483	–					
2. Cop-avoidant	15.824	7.384	0.598***	–				
3. Cop-approach	30.781	7.577	−0.016	0.138***	–			
4. Cop-humor	4.048	2.492	−0.091*	0.166***	0.259***	–		
5. Cop-religion	3.277	2.627	0.139***	0.183***	0.239***	−0.019	–	
6. Humor	15.627	5.115	−0.325***	−0.123**	0.235***	0.473***	−0.055	–

*p < 0.05, **p < 0.01, ***p < 0.001. The variables were reported as follows: stress, perceived stress; cop-avoidant, avoidant coping strategy; cop-approach, approach coping strategy; cop-humor, humor coping strategy of the Brief-COPE scale; cop-religion, religion coping strategy; humor, humor coping strategy of the CHS scale.

We performed an analysis using an ANOVA table on a linear model after analyzing four diagnostic plots (“residual vs fitted plot,” “normal Q–Q plot,” “scale-location plot,” and “residual vs leverage plot”) for regression analysis.

The linear model was selected via a hierarchical procedure (Raudenbush and Bryk, 2002; Gelman and Hill, 2007; Cohen, 2013) in which seven different models (for each dependent variable) of increasing complexity were built and compared in terms of goodness of fit.

Lastly, to further investigate the relationship between health professionals’ coping humor and exposure to risk, we performed a second analysis using an ANOVA table on a simple linear model, where “worked with COVID-19” and “COVID-19 contracted” were used as the predictors and CHS score was used as the dependent variable. Here also, the diagnostic plots were analyzed.

The *post hoc* tests used the Bonferroni correction. The Cohen’s *d* was reported as a measure of effect size. There were no missing answers. The level of significance was *p* < 0.05.

## Results

The results of our analyses support the hypotheses that HCWs’ COVID-19-related perceived stress varies in relation to different types of risk exposure (i.e., having had any contact with COVID-19 patients or not) and that HCWs who used more coping humor and approaching coping strategies instead of avoidant coping strategies perceived less COVID-19-related stress. Concerning the latter, avoidant coping and humor coping strategies were observed to be differently associated with perceived stress. However, no significant association was found between using coping humor to face stress and exposure to risk. Thus, the use of humor to cope with stressors was found to be independent of HCWs’ exposure to COVID-19.

Specifically, the results of the likelihood ratio test calculated between the complex models revealed that model no. 6 showed the best overall fit and the maximum significant reduction in the residual sum of squares [see Table 3; perceived stress ∼ worked with COVID-19 × (cop-avoidant + cop-approach + cop-humor + cop-religion + humor)].

**TABLE 3:** Likelihood ratio tests on perceived stress.

**Model (dependent variable = perceived stress)**	**RSS**	**df**	***F***	***p*-value**	**AIC**	**BIC**	***R*^2^**	**RMSE**	**Ranking**
1. Cop-avoidant	30,863				4,216.901	4,230.221	0.311	7.033	7
2. Mod1 + cop-approach	29,924	1	21.957	<0.001	4,199.582	4,217.333	0.333	6.922	6
3. Mod2 + cop-humor	28,633	1	30.166	<0.001	4,174.021	4,196.201	0.360	6.773	4
4. Mod3 + cop-religion	28,505	1	2.975	0.085	4,173.233	4,199.865	0.362	6.752	5
5. Mod4 + humor	26,875	1	38.104	<0.001	4,138.410	4,217.714	0.403	6.563	2
6. Mod5* (worked with CODIV-19)	25,655	1	2.374	0.005	4,133.404	4,169.482	0.433	6.411	1
7. Mod6* (COVID-19 contracted)	24,608		0.764	0.822	4,171.341	4,397.671	0.450	6.283	3

*RSS, reduction in the residual sum of squares; df, degree freedom; *F*, *F*-test result; *p*-value, *p*-value of the *F*-test; AIC, Akaike’s information criterion; BIC, Bayesian information criterion; *R*^2^, *R*-squared value; RMSE, root mean squared error; in the column “Ranking,” the lower number value indicates the model with better performance in terms of model fit. The ranking number is an exploratory index based on the mean value of all indices for each model. The variables were reported as follows: cop-avoidant, avoidant coping strategy; cop-approach, approach coping strategy; cop-humor, humor coping strategy of the Brief-COPE scale; cop-religion, religion coping strategy; humor, humor coping strategy of the CHS scale; worked with COVID-19, health professionals who had worked with COVID-19 patients; COVID-19 contracted, health professionals who may had contracted the virus.*

Supplementary Figure 1 reports the diagnostic plots for the selected model. All prerequisites were met.

In order to evaluate the adequacy of the sample size, a statistical power analysis was conducted on a selected linear model for sample size estimation, considering that *R*-squared value was 0.429 and, consequently, the overall Cohen’s *f*^2^ effect size was 0.830 (Cohen, 1988). With an alpha = 0.05, power = 0.80, and numerator degrees of freedom of the *F*-test = 17, a sample size of approximately 40 subjects was needed. Thus, our proposed sample size of 625 subjects was adequate.

**FIGURE 1:**
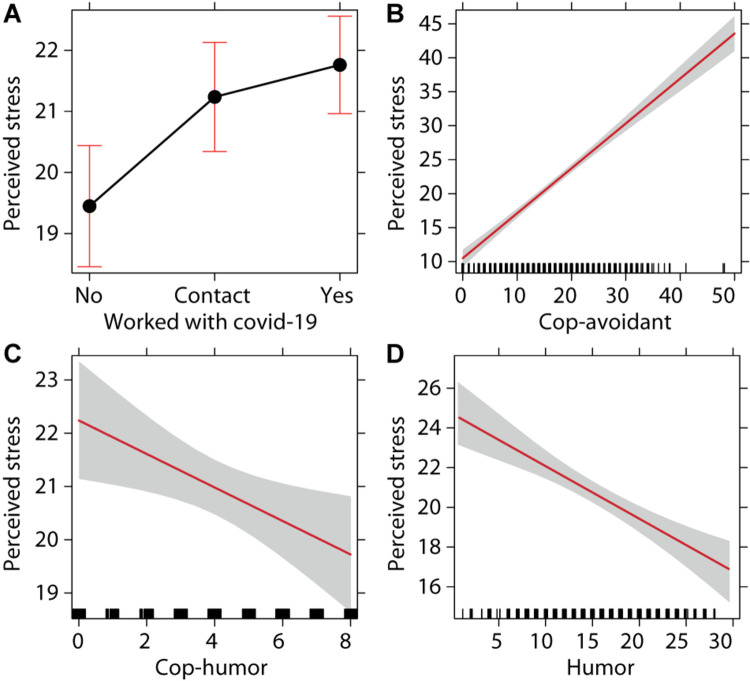
**(A)** Means and 95% confidence intervals of perceived stress in the health professionals who worked with COVID-19 patients (Yes, health professionals worked with COVID-19 patients in a dedicated ward; Contact, health professionals did not work with COVID-19 patients in a dedicated ward, but they had contacts with patients; No, health professionals did not work with COVID-19 patients). **(B)** Regression line and 95% confidence interval between cop-avoidant and perceived stress. **(C)** Regression line and 95% confidence interval between cop-humor and perceived stress. **(D)** Regression line and 95% confidence interval between humor and perceived stress.

The ANOVA performed on the selected linear model revealed a significant effect of “worked with COVID-19” variable, with *F*(2,607) = 5.067 and *p* = 0.007. The perceived stress was high for the response “yes” with *M* = 22.383, SD = 8.677, lower 95% CI = 21.308, and upper 95% CI = 23.457, compared to “contact” with *M* = 20.881, SD = 8.451, lower 95% CI = 19.712, and upper 95% CI = 22.051 and “no” with *M* = 19.076, SD = 7.864, lower 95% CI = 17.882, and upper 95% CI = 20.271. *Post hoc* analyses indicated a significant difference between “no” and “contact” (Cohen’s *d* = −0.262, *t*-ratio = −2.630, *p* = 0.026) and between “no” and “yes” (Cohen’s *d* = −0.339, *t*-ratio = −3.566, *p* = 0.001; see Figure 1A). In agreement with our hypothesis, according to which exposure to infection risk is associated with a high level of perceived stress, HCWs directly involved with COVID-19 patients showed higher stress levels than those who worked in no-COVID wards, had minimal contact, or had no contact. Those who only had indirect contact were still more stressed than those who did not have any contact.

Regarding coping strategies adopted by the respondents, a significant direct relationship was found between “cop-avoidant” and perceived stress, with *F*(1,607) = 273.516 and *p* < 0.001 (see Figure 1B). In contrast, the use of humor as a coping strategy is negatively associated against perceived stress. In fact, a significant inverse relationship was found between “cop-humor” and perceived stress, with *F*(1,607) = 4.777 and *p* = 0.029 (see Figure 1C). Moreover, a significant inverse relationship was also found between “humor” and perceived stress, with *F*(1,607) = 28.892 and *p* < 0.001 (see Figure 1D). There were no significant interaction effects (for complete data, see Table 4).

**TABLE 4:** Fixed-effects ANOVA results.

	**df**	***F***	***p***	**Partial eta-square**
Worked with COVID-19	2	5.067	0.007	0.016
Cop_avoidant	1	273.516	<0.001	0.311
Cop_approach	1	3.289	0.070	0.005
Cop_humor	1	4.777	0.029	0.008
Cop_rel	1	3.006	0.835	0.004
Humor	1	28.892	<0.001	0.045
Worked with COVID-19 × cop_avoidant	2	2.467	0.086	0.008
Worked with COVID-19 × cop_approach	2	2.371	0.094	0.007
Worked with COVID-19 × cop_humor	2	0.460	0.631	0.002
Worked with COVID-19 × cop_rel	2	0.418	0.659	0.001
Worked with COVID-19 × humor	2	0.228	0.797	<0.001
Residuals	607			

*The variables were reported as follows: cop-avoidant, the avoidant coping strategy; cop-approach, the approach coping strategy; cop-humor, the humor coping strategy of the Brief-COPE scale; cop-religion, the religion coping strategy; humor, the coping strategy of the CHS scale; worked with COVID-19, the health professionals who had worked with COVID-19 patients; COVID-19 contracted, the health professionals who may had contracted the virus. Perceived stress is the dependent variable.*

Regarding the linear model between health professionals’ coping humor and exposure to risk, diagnostic plots are reported in Supplementary Figure 2. All prerequisites were met.

The power analysis was conducted (*R*-squared = 0.0327, Cohen’s *f*^2^ effect size = 0.034, alpha = 0.05, power = 0.80, numerator degrees of freedom of the *F*-test = 8). The sample size needed was approximately equal to 452. Thus, our sample size was adequate.

The ANOVA performed on this model revealed no significant main effects and interactions (all *p* > 0.07).

## Discussion

The COVID-19 pandemic has posed great challenges to HCWs worldwide. This study investigated the psychological impact of the pandemic on HCWs’ wellbeing by focusing on their perceived stress during the outbreak, the role played by their exposure to COVID-19, approaching vs avoidant coping strategies, and coping humor. The findings predominantly suggest that perceived stress was affected by HCWs’ exposure to COVID-19 and use of avoidant coping and humor-based coping strategies. HCWs who reported higher levels of perceived stress were more exposed to COVID-19, as they had worked in COVID-19-dedicated wards and/or had come into contact with infected people. These results appear partially different from those obtained by Rossi et al. (2020) on a sample of Italian HCWs, according to which a high level of perceived stress was associated with having a deceased, hospitalized, or quarantined colleague rather than being exposed to contagion (which was instead associated with a high level of depression). Nonetheless, the findings seem to be consistent, for example, with the findings obtained by Du et al. (2020), according to which the perceived stress among frontline HCWs in Wuhan during the outbreak ranged from moderate to severe, and those of Lai et al. (2020), according to which Chinese frontline HCWs (i.e., workers directly engaged in the diagnosis, treatment, and care of COVID-19 patients) reported high levels of psychological burden.

Regarding coping strategies, participants who reported a higher use of avoidant coping strategies perceived the situation as more stressful than those who used them less. This outcome agrees with previous studies on Italian rescue workers, which included analysis on healthcare professionals (Prati et al., 2011), as well as on Chinese students during the COVID-19 outbreak (Ye et al., 2020). In addition, participants who reported a higher use of humor-based coping strategies perceived the situation as less stressful in comparison with those who reported lower levels of coping humor. This result extends to the healthcare setting of Martin et al.’s (1993) study, which supported the positive impact of coping humor on perceived stress (both assessed through the same scales we used: CHS and PSS) of students facing academic examinations.

As a general outcome, humor was positively associated with the wellbeing of health workers; therefore, it could reduce perceived stress related to COVID-19, thanks to the positive influence of a humorous mood on cognitive assessment and re-evaluation of the stressful situation (Martin et al., 1993; Abel, 2002; Doosje et al., 2012). This outcome adds to previous literature showing the protective power of humor against life-threatening and stressful situations (e.g., Vaillant, 2000; Peterson and Seligman, 2004; Geisler and Weber, 2010; Müller and Ruch, 2011; Kuiper, 2012), including daily stressful situations within an HCW’s work life (Plester, 2009; Rowe and Regehr, 2010). The evidence from this study suggests that when coping humor strategies are adopted, the HCWs’ perceived stress might decrease even in an extraordinarily critical situation, such as a pandemic. Interestingly, the use of humor to cope with stressors was found to be independent of HCWs’ exposure to COVID-19. This outcome is supported by previous literature showing that a sense of humor, which includes its coping function (Martin, 1996; McGhee, 1999), is a disposition relatively stable over time and situations (Martin, 1996; McGhee, 1999; Ruch, 2008) and is even considered as a trait in Ruch et al.’s (1996) temperamental approach to humor.

As indirect proof that this disposition is at work also in times of COVID-19, several jokes, humorous memes, and short videos targeting the impact of the pandemic on social, political, economic, and everyday life have been circulating on the Internet and social media (Facebook, Twitter, etc.) during the pandemic worldwide (Bischetti et al., 2020; Jimenez-Sotomayor et al., 2020). In fact, even dark humor has been conceptualized as a coping strategy, at least in a healthcare context (Plester, 2009). Obviously, not all of them have the positive function of buffering difficulties and helping people cope (Jimenez-Sotomayor et al., 2020). However, the ability to cope with difficulties via humor (assessed through CHS), associated with the personal disposition toward optimism, predicted ratings of funniness and aversiveness toward COVID-19 humorous stimuli in a large sample of Italian people (*N* = 1,751; Bischetti et al., 2020). In particular, the more the participants reported using humor as a coping strategy, the more they rated COVID-19 humorous stimuli as funny. Conversely, those who reported lower levels on CHS perceived the same COVID-19 humorous stimuli as adverse. Similarly, Saroglou and Anciaux (2004) found that sick-humor appreciation is positively related to coping humor (measured via Brief-COPE Scale). Moreover, Bischetti et al. (2020) also found that psychological distancing (measured in terms of perceived risk of being infected, spatial distancing from the epicenter of COVID-19 outbreak in Italy, and social distancing in terms of number of relatives or friends infected with COVID-19) impacted humor appreciation: participants with a distanced perspective appreciated COVID-19 humorous stimuli, whereas those with an immersive perspective judged them as aversive (Bischetti et al., 2020). Conversely, psychological distancing, though differently operationalized, had no relation with the use of coping humor in the sample we surveyed. In fact, in our study, we investigated HCWs’ exposure to COVID-19 by asking participants whether they had worked in wards dedicated to COVID-19, had come into contact with COVID-19 patients, or were infected with COVID-19; these items at least partially address the issue of psychological distancing, as investigated in the field of dark and disaster humor (Kuipers, 2006; Morreall, 2009; McGraw et al., 2012, 2013; Bischetti et al., 2020). Overall, these results suggest that appreciation of dark humor is affected by psychological distancing and coping humor, which work independent of each other; however, till date, their magnitude on humor appreciation remains unexplored but worth investigating. Psychological distancing is a multifaceted concept, sometimes used with different shades of meanings, which, owing to its centrality in appreciation of dark and disaster humor, would need to be framed in a global perspective, accounting for its types and extensions within the domain of humor studies. However, this falls outside the aims of our study, which hopefully could contribute to the debate on psychological distancing in humor.

### Limitations and Future Studies

An important limitation of our study is that we did not deepen the emotional impact of stressors on HCWs and how coping humor contributed to managing their emotional reactions and balance. However, several studies have pointed out that coping humor positively impacts managing and softening of HCWs’ negative emotions and promotes positive emotions. For example, humor intervention programs enhance subjective wellbeing (Ruch and McGhee, 2014; Wellenzohn et al., 2018; Tagalidou et al., 2019); positive humor decreases anxiety, perceived stress, and depression (Crawford and Caltabiano, 2011) and is associated with emotional regulation and job satisfaction (Plester, 2009; Sun et al., 2017). Although positive humor regulates emotions better than negative humor (Samson and Gross, 2012), even negative humor used within the healthcare context (i.e., gallows humor or black humor) has the protective functions of creating social support with colleagues and distancing from their own and patients’ negative emotions measured by illness, death, and human suffering in order to act sharp wittedly (Rowe and Regehr, 2010). Further studies may focus on the role played by coping humor in fostering emotion regulation and emotional balance of HCWs facing pandemic stressors.

### Practical Implications

Follow-up studies on the SARS pandemic revealed that HCWs’ high levels of work-related distress, burnout, maladaptive behaviors, and coping strategies were often long-lasting, ranging up to 2 years after outbreak resolution (Maunder et al., 2006). Identifying hospital-based interventions to promote and support HCWs’ mental health and wellbeing is an urgent requirement for healthcare systems and workplaces. Moreover, recent neurobiological studies (Efstathopoulos et al., 2018; McLoughlin et al., 2020) related to the NR31C1 factor, the glucocorticoid receptor gene, have shown that stress has a decisive impact on one’s future: stress inhibits the ability to find positive coping strategies and empathy with others and reduces the energy used in emergency and/or learning situations, including the possibility of recognizing potential dangers. Therefore, the positive impact of humor on stress inhibition could have positive and long-term preventive effects both on HCWs’ professionalism and on their relationship with patients. Overall, our study adds new evidence to research, revealing that coping humor could be an effective strategy that contributes to a wide array of psychological outcomes, including stress perception mitigation.

The positive impact of coping humor might suggest the appropriateness of interventions of humor-based protocols for supporting HCWs’ wellbeing, given that their positive effects on mental health protection have largely been demonstrated (Crawford and Caltabiano, 2011; Ruch and McGhee, 2014; Wellenzohn et al., 2018; Tagalidou et al., 2019). Moreover, individuals who use avoidant strategies to cope with difficult situations appraise these situations as threatening (Carver and Scheier, 1994; Dias et al., 2012), whereas individuals inclined toward coping humorously with difficult situations appraise them as more challenging and less threatening than those who do not rely on coping humor (Martin et al., 1993). Therefore, humor-based interventions for HCWs may provide them with preventive tools to appraise a problematic circumstance as challenging. This could be a key to reducing the use of avoidant coping strategies, which suggested to be a maladaptive strategy in our study owing to their negative impact on HCWs’ stress perception.

## Data Availability Statement

The original contributions presented in the study are included in the article/Supplementary Material, further inquiries can be directed to the corresponding author/s.

## Ethics Statement

The studies involving human participants were reviewed and approved by Ph.D. meeting curriculum in Psychology, Communication and Social Sciences, University of Macerata, project identification code n. 19435, August 3, 2020. The patients/participants provided their written informed consent to participate in this study.

## Author Contributions

CC contributed to the conceptualization, investigation, writing—original draft preparation (Abstract, Introduction, and Discussion), review and editing, supervision, and project administration. RBo, AF, and AB contributed to the conceptualization, investigation, and writing—original draft preparation (Materials and Methods). IR and MM contributed to the conceptualization, investigation, review, and editing. RBu contributed to the conceptualization, data curation, formal analysis, investigation, data analysis, supervision, and writing—original draft preparation (Materials and Methods, Data Analysis, and Results). All authors contributed to the article and approved the submitted version.

## Conflict of Interest

The authors declare that the research was conducted in the absence of any commercial or financial relationships that could be construed as a potential conflict of interest.

## Footnotes

1 https://covid19.who.int/, retrieved on November 24, 2020.

2 Ministero della salute: http://www.salute.gov.it, retrieved on July 23, 2020.

3 Governo Italiano, Presidenza del Consiglio dei Ministri: http://www.governo.it/sites/new.governo.it/files/DPCM4MARZO2020.pdf, retrieved on July 23, 2020.

4 Specifically, two of the authors separately translated the English version into Italian; then, they compared their translations and reached an agreement on a single version. This version was back-translated by a colleague (fluent in Italian) who is a native English speaker; the colleague knew neither the aims of the research nor the original scale. Her version was compared with the original one. Since the two versions were very similar, the translation was taken into use.
