# Supplementary material for: Coronavirus Disease Stress Among Italian Healthcare Workers: The Role of Coping Humor
Source: Front Psychol. 2021 Jan 25;11:601574. doi: 10.3389/fpsyg.2020.601574 (PMC7868596; doi:10.3389/fpsyg.2020.601574)
Supplement: Supplementary file 1 [file Data_Sheet_1.docx]

Supplementary Material

## Supplementary Figures


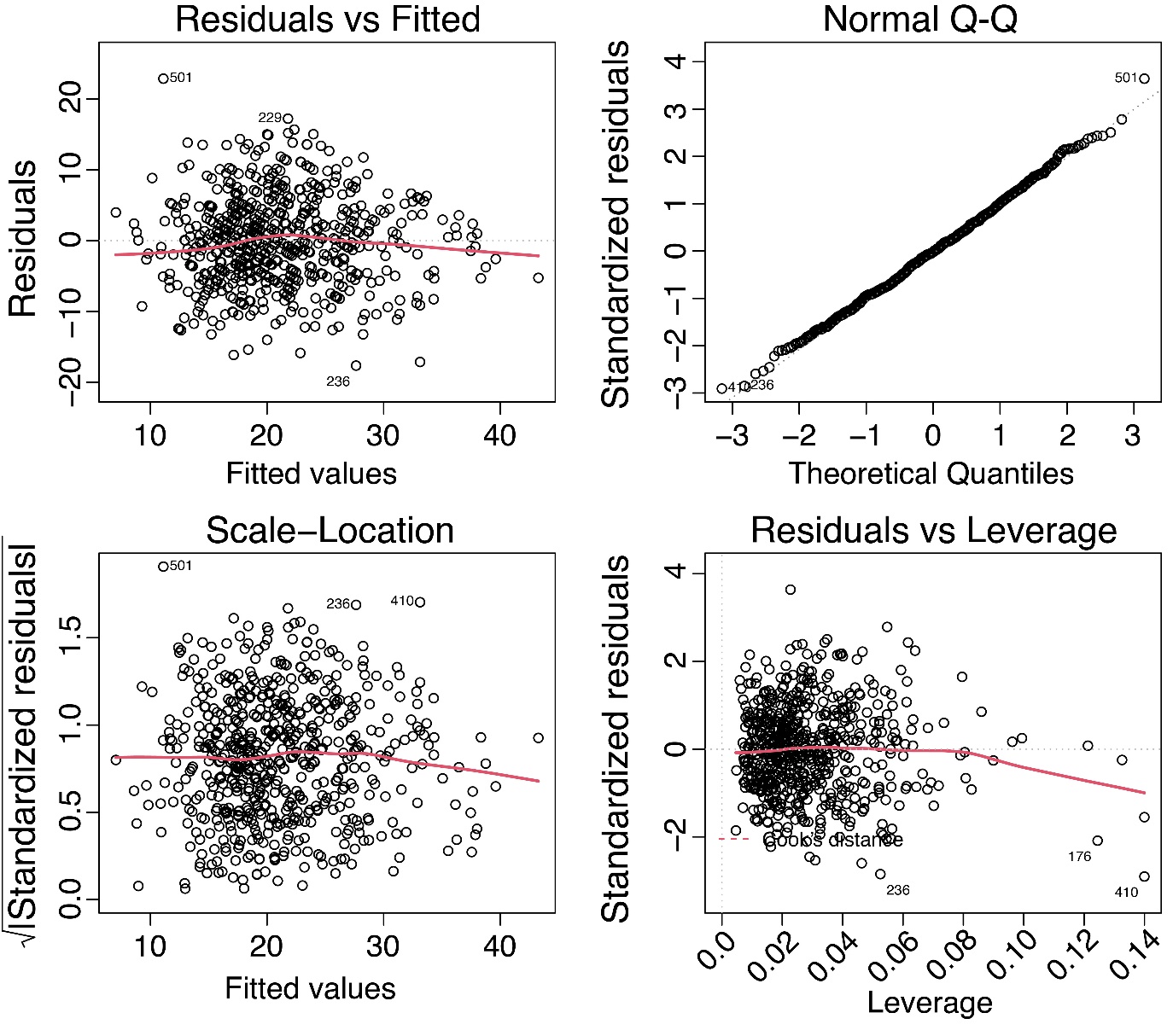


**Supplementary Figure S1.** The diagnostic plots for the selected linear model.


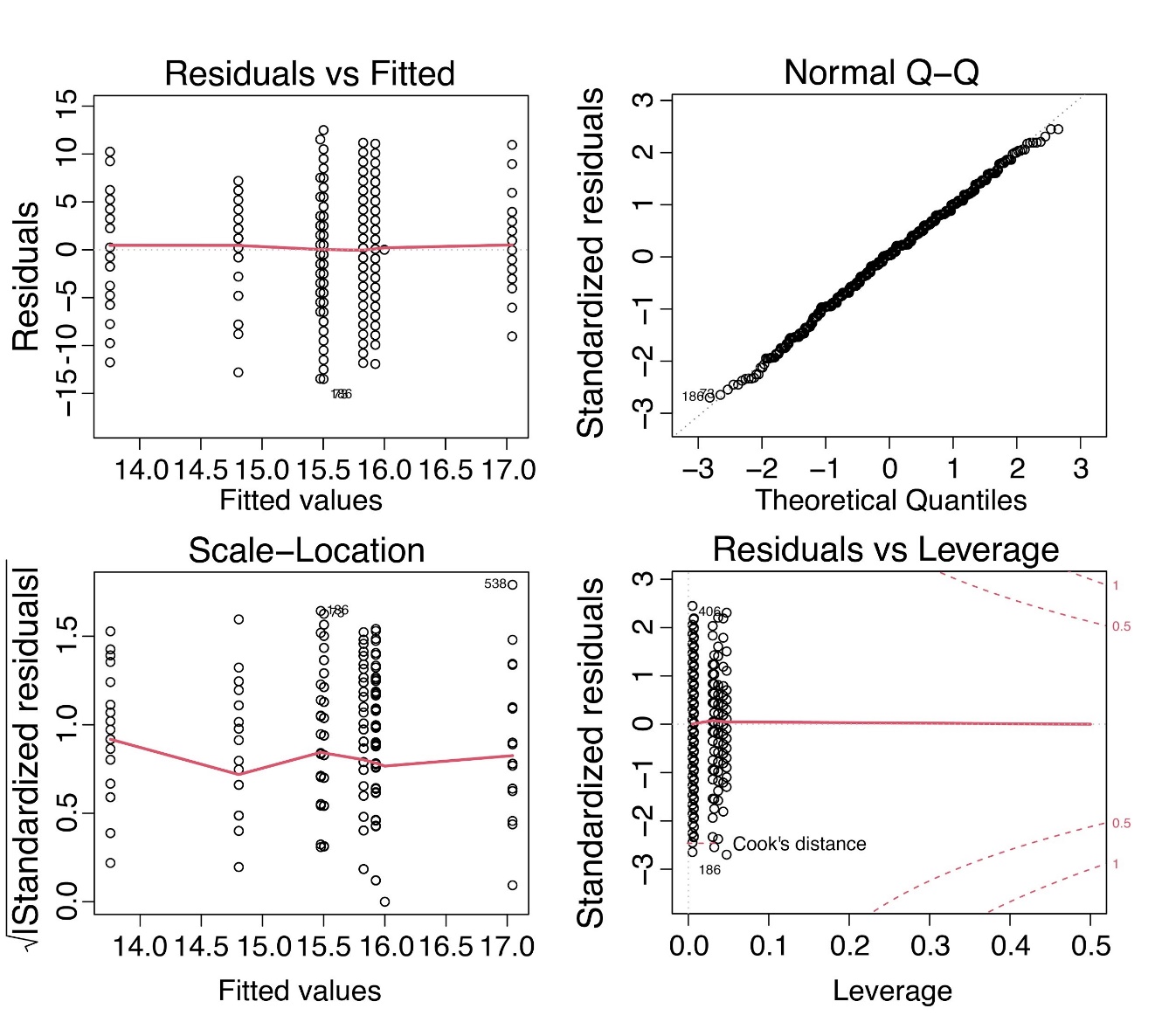


**Supplementary Figure S2.** The diagnostic plots for the linear model between health professionals’ coping humor and exposure to risk.
